# Hereditary Angioedema Nationwide Study in Slovenia Reveals Four Novel Mutations in *SERPING1* Gene

**DOI:** 10.1371/journal.pone.0056712

**Published:** 2013-02-20

**Authors:** Matija Rijavec, Peter Korošec, Mira Šilar, Mihaela Zidarn, Jovan Miljković, Mitja Košnik

**Affiliations:** 1 University Clinic of Respiratory and Allergic Diseases Golnik, Golnik, Slovenia; 2 Department of Dermatovenerology, University Clinical Centre Maribor, Maribor, Slovenia; Tor Vergata University of Rome, Italy

## Abstract

Hereditary angioedema (HAE) is a rare autosomal dominant disease characterized by swelling of the face, lips, tongue, larynx, genitalia, or extremities, with abdominal pain caused by intra-abdominal edema. HAE is caused by mutations affecting the C1 inhibitor gene, *SERPING1*, resulting in low levels of C1 inhibitor (Type I HAE) or normal levels of ineffective C1 inhibitor (Type II HAE). A nationwide survey identified nine unrelated families with HAE in Slovenia, among whom 17 individuals from eight families were recruited for genetic analyses. A diagnosis of HAE was established in the presence of clinical and laboratory criteria (low C1 inhibitor antigenic levels and/or function), followed up by a positive family history. Genetic studies were carried out using PCR and sequencing to detect *SERPING1* mutations in promoter, noncoding exon 1, the 7 coding exons, and exon-intron boundaries. Multiplex ligation-dependent probe amplification was performed in order to search for large deletions/duplications in *SERPING1* gene. A mutation responsible for HAE was identified in patients from seven families with the disease. In HAE type I families, one previously reported substitution (Gln67Stop, c.265C>T) and four novel mutations were identified. The new mutations included two missense substitutions, Ser128Phe (c.449C>T), and Glu429Lys (c.1351G>A), together with two frameshift mutations, indel (c.49delGinsTT) and deletion (c.593_594delCT). Both families with HAE type II harbored the two well-known substitutions affecting the arginyl residue at the reactive center in exon 8, Arg444Cys (c.1396C>T) and Arg444His (c.1397G>A), respectively. In one patient only the homozygous variant g.566T>C (c.-21T>C) was identified. Our study identified four novel mutations in the Slovenian HAE population, highlighting the heterogeneity of mutations in the *SERPING1* gene causing C1 inhibitor deficiency and HAE. In a single patient with HAE a homozygous variant g.566T>C (c.-21T>C) might be responsible for the disease.

## Introduction

Hereditary angioedema (HAE) is a rare autosomal dominant disease, with an estimated prevalence of one case per 50,000 persons, characterized by swelling of the face, lips, tongue, larynx, genitalia, or extremities, with abdominal pain caused by intra-abdominal edema [1]–[4]. HAE is caused by mutations affecting the C1 inhibitor gene, *SERPING1*, resulting in low levels of C1 inhibitor (Type I HAE) or by normal levels of ineffective C1 inhibitor (Type II HAE) [1]–[3]. C1 inhibitor is the inhibitor of the first component of the complement system, thus preventing the inappropriate or excessive activation of the complement system. In addition to classical pathways, C1 inhibitor also controls the lectin complement pathway [1], [2]. Furthermore, it controls the mannose-binding protein-associated serine protease system, as well as kallikrein, coagulation factors XIIa and XIa, plasmin, and tissue plasminogen activator [1]. C1 inhibitor is therefore a key regulator of several immune and inflammatory pathways. Estrogen-dependent or HAE type III occurs in patients with normal C1 inhibitor level and function. It mainly involves women and the exact mechanism remains unknown, although in some patients a mutation in the coagulation factor XII gene have been identified [1], [2].

The *SERPING1* gene is located in the q12–q13.1 subregion of chromosome 11, and it consists of eight exons and seven introns distributed over 17 kb, with introns containing 17 repetitive *Alu* sequences [1], [5]. To date more than 250 different mutations in *SERPING1* have been described in the HAE database, ranging from nucleotide substitutions and small insertions and deletions to large deletions and duplications (HAEdb, http://www.hae.enzim.hu) [6], resulting in low levels of C1 inhibitor (85% of cases) or in normal levels of C1 inhibitor with low functional activity (15% of cases) [1], [7]–[21]. De novo mutations in *SERPING1* account for about 25% of cases with HAE [1], [9]. Due to high *SERPING1* gene variability, studies focusing on genetic epidemiology in different populations are needed to identify novel *SERPING1* mutations, which would also provide new insight into the structure-function relationship.

## Materials and Methods

### Patients

The University Clinic of Respiratory and Allergic Diseases Golnik is the only tertiary allergy hospital in Slovenia and is responsible for nationwide HAE diagnosis and treatment. A nationwide survey identified nine unrelated families with HAE in Slovenia. We recruited 17 patients with HAE from eight unrelated Slovenian families for genetic analysis (one family refused to participate in this study). The diagnosis of HAE was established in the presence of at least one major clinical criterion (subcutaneous angioedema, abdominal pain, laryngeal edema) and one laboratory criterion (C1 inhibitor antigenic levels, C1 inhibitor function), followed up with positive family history, as proposed in guidelines for the diagnosis of HAE [1], [2]. The study was approved by the state ethics committee and all participants gave their informed written consent.

### Clinical Severity Score

The clinical severity score was calculated based on the age of disease onset (0–5 years  = 3 points, 6–10 years  = 2 points, 11–20 years  = 1 point, >20 years  = 0 points), number of organs affected (skin edema  = 1 point, painful abdominal edema  = 2 points, laryngeal edema  = 2 points, other clinical manifestations  = 1 point) and need for long-term prophylaxis (long term prophylaxis  = 1 point), and expressed with values from 0 to 10 as proposed by Bygum et al. [19].

### Complement Testing

Serum protein concentrations of C1 inhibitor (normal range: 0.20–0.35 g/l), C4 (normal range: 0.16–0.31 g/l) (Siemens, Marburg, Germany), and C1q (The Binding Site, Birmingham, UK) were quantified by means of radial immunodiffusion and C1 inhibitor function (C1 inhibitor functional levels ≤40% of normal are considered decreased) were measured using an enzyme immunoassay (Quidel Corporation, California, USA) in accordance with the manufacturer's instructions.

### Genotyping

Genomic DNA was extracted from EDTA-containing whole blood samples using a QIAamp DNA Blood Mini Kit (Qiagen, Hilden, Germany) according to the manufacturer’s instructions. The detection of *SERPING1* mutations in promoter, noncoding exon 1, the 7 coding exons and exon-intron boundaries were performed as described previously [3], [17]. To identify mutations, all sequences were compared with the *SERPING1* reference sequence in the GenBank (GenBank accession number X54486.1). *SERPING1* variations were numbered in two ways. The traditional genomic numbering considers the first nucleotide of exon 1 to be number one [5], whereas the systematic cDNA numbering considers the first nucleotide (A) of the initiation methionine (ATG) of the cDNA sequence (GenBank accession number NM_000062.2) to be nucleotide number one. For the protein amino acid positions, the study used traditional numbering based on the mature protein of 478 amino acids, counting the first 22 amino acids of the N-terminal residue of the signal peptide in negative numbers. In order to search for large deletions/duplications multiplex ligation-dependent probe amplification (MLPA) was performed using the SALSA MLPA P243-A2 SERPING1 kit (MRC-Holland, The Netherlands) and data were analyzed with GeneMapper software v4.0 (Applied Biosystems, Foster City, California, USA).

## Results

### Clinical Details

Clinical and laboratory data are presented in Table 1. Eleven patients from five families were diagnosed with HAE type I, and six patients from three families with HAE type II. The mean C1 inhibitor function was 21% (range 1–64%). HAE type I patients have highly reduced concentrations of C1 inhibitor in serum, whereas patients with HAE type II have normal or raised levels of C1 inhibitor (mean 0.44 g/l).

**Table 1:** Clinical features of Slovenian patients with HAE.

Family	Age (years)	Gender	Age at onsetof symptoms	HAE diagnosis (type)	Clinicalseverity score	Skin oedema	Facial oedema	Abdominal oedema	Laryngeal oedema	Prophylactic treatment	Family history	C1-INH, g/l	C1-INH function, %	C4, g/l
1	28	M	3	Type I	10	+	–	–	–	Danazol	+	0.09	19	0.15
1	62	M	25	Type I	5	+	–	–	–	None	+	<0.05	26	0.09
1	83	F	ND^1^	Type I	ND	+	+	–	–	None	–	0.06	53	0.14
2	38	F	21	Type I	5	+	+	+	+	Danazol	+	0.05	4	0.09
2	73	M	Asympt	Asympt	ND	–	–	–	–	None	+	<0.05	26	0.05
3	19	F	13	Type II	2	+	–	–	–	None	+	0.56	28	<0.05
3	23	M	12	Type II	2	+	–	–	–	None	+	0.59	17	<0.05
3	47	M	17	Type II	4	+	+	–	+	Danazol	+	0.66	15	0.09
4	42	M	20	Type I	4	+	+	+	–	None	+	<0.05	1	<0.05
4	77	F	21	Type I	5	+	–	–	+	None	+	<0.05	32	<0.05
5	49	F	19	Type I	7	+	–	–	–	None^2^	+	<0.05	1	0.06
5	30	M	16	Type I	6	+	–	–	+	Danazol	+	<0.05	19	<0.05
5	84	F	ND^1^	Type I	ND	ND	ND	ND	ND	None	–	<0.05	1	<0.05
6	44	M	34	Type II	6	+	+	+	+	None	+	0.25	64	<0.05
6	63	F	7	Type II	5	+	+	+	–	None	+	0.31	9	0.13
7	40	F	16	Type I	7	+	+	+	+	Danazol	+	0.08	33	<0.05
7	65	M	10	Type I	7	+	+	+	+	Danazol	–	0.06	22	0.05
8	41	F	40	Type II	1	–	+	–	–	Danazol	–	0.25	11	0.15

Asympt: Asymptomatic; ND: Not determined.

1 Patients have reported angioedema attacks in past but exact time, intensity and onset is unknown.

2 Side effects of Danazol use.

The mean age at onset of clinical symptoms was 18 years (range 3–40 years). Major symptoms were skin edema in 15 patients (88%), followed by facial edema in 9 patients (53%), laryngeal edema in seven (41%), and abdominal edema in six (35%). Attacks often occurred spontaneously; however, several triggering factors have been identified, including trauma, stress, infections, and sudden changes in temperature. The majority of patients reported few angioedema episodes annually, two patients reported attacks only in the past, and one patient was asymptomatic. Seven (41%) patients are receiving long-term prophylaxis with attenuated androgen danazol. Four patients (24%) had no known family history of angioedema.

### Genetic Analysis

**Figure 1:**
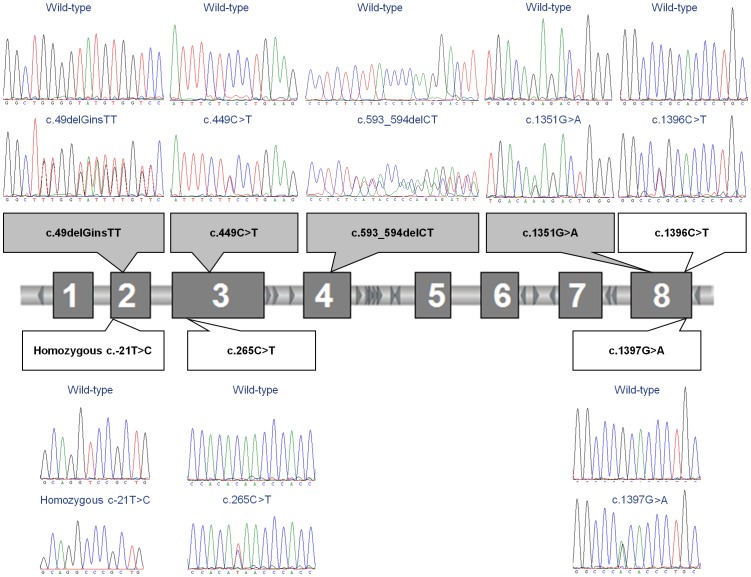
Schematic representation of *SERPING1* gene and the mutations identified in Slovenian families with hereditary angioedema. Boxes showing novel mutations identified are shadowed in grey.

In 16 patients with HAE from seven families, a mutation in *SERPING1* responsible for the disease distinct for each family was identified, whereas no mutations were present in healthy relatives and controls. Three families carried known mutations, whereas four mutations were reported for the first time (Table 2 and Figure 1). In five HAE type I families, one already reported mutation in exon 3 (Gln67Stop, c.265C>T), and four novel mutations have been identified. The novel mutations included two missense substitutions, one in exon 3 Ser128Phe (c.449C>T) and the second in exon 8 Glu429Lys (c.1351G>A), together with two frameshift mutations: one combination of a deletion and insertion (“indel”) in exon 2 (c.49delGinsTT) and one deletion in exon 4 (c.593_594delCT). The two novel missense substitutions, Ser128Phe (c.449C>T) and Glu429Lys (c.1351G>A), are predicted as damaging with high confidence levels by the PolyPhen-2 (http://genetics.bwh.harvard.edu/pph2/) and SIFT (http://sift.jcvi.org/) programs. The PolyPhen-2 scores of c.449C>T and c.1351G>A were 0.990 and 1.000, whereas the SIFT scores were 0.00 and 0.01, respectively. Furthermore, the family with HAE harboring the c.449C>T mutation, consisted of two affected family members with c.449C>T mutation, whereas this mutation was absent in a healthy relative without any symptoms and with normal C1 inhibitor level and activity. In a family harboring the c.1351G>A missense substitution, one patient was symptomatic, whereas the other patient carrying this mutation was asymptomatic, but both patients have reduced C1 inhibitor level and activity. Asymptomatic adults with *SERPING1* mutations are estimated to account for approximately 5% of all patients with HAE [1]. On the other hand, this mutation was absent in several unaffected family members with normal C1 inhibitor levels and activity. These data, together with the fact that no mutations were present in healthy controls, strongly suggest that the identified mutations are indeed responsible for the disease.

**Table 2:** Mutations found in Slovenian patients with HAE.

Family	Traditional genomic numbering	cDNA numbering	Exon	Predicted effect on protein (traditional numbering)	Reference
1	g.2409C>T	c.265C>T	3	Gln67Stop	16
**2**	**g.16743G>A**	**c.1351G>A**	**8**	**Glu429Lys**	**this study**
3	g.16788C>T	c.1396C>T	8	Arg444Cys	7
**4**	**g.2593C>T**	**c.449C>T**	**3**	**Ser128Phe**	**this study**
**5**	**g.635delGinsTT**	**c.49delGinsTT**	**2**	**frameshift**	**this study**
6	g.16789G>A	c.1397G>A	8	Arg444His	7
**7**	**g.4393_4394delCT**	**c.593_594delCT**	**4**	**frameshift**	**this study**
8	g.566T>C^1^	c.-21T>C^1^	2	Possible splicing defect	8,13,16,19

New mutations are in boldface type.

1 Homozygous nucleotide change; polymorphism referred as non-pathogenic in heterozygous form.

Mutations c.49delGinsTT and c.593_594delCT cause frameshift and premature stop at two and 57 amino acids downstream, respectively. Furthermore, both families harboring frameshift mutations consisted of several patients with HAE; specifically, three affected family members with c.49delGinsTT and two affected family members with c.593_594delCT. In unaffected relatives or controls, these mutations were absent, confirming that the identified mutations are indeed responsible for the disease.

Both families with HAE type II harbored the two well-known substitutions affecting the arginyl residue at the reactive center in exon 8, Arg444Cys, c.1396C>T and Arg444His, c.1397G>A, respectively. Those patients had a normal C1 inhibitor level but reduced activity because mutations at this position alter the target protease recognition site of this protein. Three affected family members carrying the c.1396C>T substitution and two affected family members carrying the c.1397G>A substitution, were identified.

In one patient with clinical symptoms of HAE and reduced C1 inhibitor activity, no mutation responsible for the disease, except the homozygous variant g.566T>C (c.-21T>C), was identified. This change is located in the non-translated region and was previously referred to as a non-pathogenic polymorphism in heterozygous form [8], [13], [16], [19], but might be pathogenic in homozygous form [22]. In this patient large deletions/duplications in *SERPING1* gene have been excluded with the use of MLPA.

## Discussion

In our cohort of eight Slovenian families with a clinical diagnosis of HAE, mutations in the *SERPING1* gene responsible for the disease were identified in all patients from seven families. In one patient no mutation was identified, with the exception of the homozygous variant g.566T>C (c.-21T>C).

The prevalence of HAE is estimated to be approximately one case per 50,000 persons without major ethnic or gender differences [2], [4]. The Slovenian prevalence of symptomatic patients is 1∶105,000, which is similar to what is reported in Spain [14], [23], and slightly lower than reported in a Danish nationwide study, in which the prevalence was reported to be 1∶71,000 [19], [24], suggesting that this rare disease may still be under-diagnosed.

The Slovenian cohort consisted of five families with HAE type I (63%) and three families with HAE type II (37%). The frequency of HAE type I is lower than previously reported, whereby the reported frequencies in studies from other European countries, such as Spain, Germany, Italy, Denmark and Hungary, were between 80 and 92% [13]–[16], [19], [21], [23], [25]. However, a study performed on patients of Czech origin with HAE reported on five families with HAE type I and four families with HAE type II [12], which is similar to our findings. Our results may indicate that the frequency of different HAE types (I and II) might differ between patients of different origin.

Mutations responsible for the disease in HAE type I were identified in all patients and were distributed across several exons, specifically exon 2, 3, 4 and 8 (Table 2 and Figure 1). One nonsense substitution in exon 3 (Gln67Stop, c.265C>T) has already been reported [16], whereas four novel mutations, which included two missense substitutions, Ser128Phe (c.449C>T) and Glu429Lys (c.1351G>A), and two frameshift mutations, c.49delGinsTT and c.593_594delCT, have been identified. Frameshift mutations are expected to alter the reading frame or lead to a premature termination of the protein, and as a result those unstable mRNA transcripts are removed through the nonsense-mediated mRNA decay pathway [26].

Both families with HAE type II harbored the two well-known substitutions affecting the arginyl residue at the reactive center in exon 8 [7], [10], [12]–[16], [18]–[21]. Those patients had a normal C1 inhibitor level but reduced activity because mutations at this position alter the target protease recognition site of this protein.

In a single patient with clinical symptoms compatible with HAE, relatively late symptom onset (40 years), and reduced C1 inhibitor activity, no mutation responsible for the disease, except the homozygous variant g.566T>C (c.-21T>C), was identified. This patient is receiving long-term prophylaxis with attenuated androgen danazol, and the frequency of edema attacks has been diminished with therapy. She was also supplied with C1 inhibitor concentrate and responded well to the administration during angioedema attacks. Variant c.-21T>C is the second nucleotide of exon 2, located in the non-translated region; it is part of the canonical acceptor site and may affect message splicing but not protein structure [8], [27]. This change was previously referred to as a non-pathogenic polymorphism in heterozygous form [8], [13], [16], [19], but might be pathogenic in homozygous form [22]. The frequency of heterozygous c.-21T>C was previously reported to be similar in patients with HAE and in healthy persons, ranging from 4 to 15% [8], [13], [16], [19], [27]. However in our cohort it was present only in the patient mentioned above in homozygous form, whereas it was not detected in any other patient or healthy control. Its heterozygous variant together with another mutation in the *SERPING1* gene was previously suggested to be associated with a more severe HAE form [27]; however, recently the role of this polymorphism on disease severity could not be confirmed [19]. The variation c.-21T>C possibly affects the mRNA splicing [8], [27], [28]. Furthermore, a recent report showed that the homozygous c.-21T>C variation was the only identified change in *SERPING1* in an affected patient with HAE [22]. Therefore, our findings suggest that the homozygous form of c.-21T>C in the *SERPING1* gene might be responsible for the disease. However, further studies are warranted in order to determine whether variant g.566T>C (c.-21T>C) in homozygous form is truly responsible for HAE symptoms.

To address the question of a possible correlation between different mutations and C1 inhibitor concentration or disease severity, we used a cumulative severity score questionnaire as constructed and described by Bygum et al. [19]. Compared to other widely used questionnaires, which mostly focus on the recent severity of the disease, this questionnaire focuses on a cumulative severity score and does not include items that are prone to a recall bias, such as attach frequency and severity. However, the number of patients carrying a certain mutation was too small to allow us any relevant genotype-phenotype correlation. Furthermore, in families with more affected members sharing the same mutation, different disease severity scores as well as C1 inhibitor concentrations/activities were evident. Similarly no correlations were detected between C1 inhibitor concentration/activity and disease severity, which is in line with the majority of other reports [19], [25].

In conclusion, the Slovenian cohort is characterized by a high heterogeneity of *SERPING1* mutations leading to HAE. Although there are more than 250 previously described mutations in the *SERPING1* gene (http://www.hae.enzim.hu), we have identified four novel ones together with three recurrent mutations. Due to the high frequency of *de novo* mutations in *SERPING1*, the spectrum of novel mutations will increase by genotyping of patients of different ethnic origins. Interestingly, in a single patient with HAE a homozygous variant g.566T>C (c.-21T>C) might be responsible for the disease.

The diagnosis of HAE is usually based on characteristic clinical symptoms together with measurements of C1 inhibitor level/activity. However, the detection of mutations responsible for the disease offers the possibility of early diagnosis in infants before the appearance of clinical symptoms, which is essential in the prevention and adequate treatment of life-threatening edema.
